# Development and Characterization of PBS/EA Cellulose and PCL/EA Cellulose Biocomposites: Structural, Morphological, and Thermal Insights for Sustainable Applications

**DOI:** 10.3390/polym17070971

**Published:** 2025-04-02

**Authors:** Fisokuhle Innocentia Kumalo, Moipone Alice Malimabe, Mafereka Francis Tyson Mosoabisane, Thandi Patricia Gumede

**Affiliations:** 1 Department of Chemistry, University of the Free State, QwaQwa Campus, Private Bag X13, Phuthaditjhaba 9866, South Africa; kumalofisokuhle@gmail.com (F.I.K.); mokoenama@ufs.ac.za (M.A.M.); 2 Department of Life Sciences, Central University of Technology, Bloemfontein 9301, South Africa; 3 Radiochemistry, South African Nuclear Energy Corporation Limited, Brits 0240, South Africa; mosoabisanemft@gmail.com

**Keywords:** biopolymer composites, *Eucomis autumnalis* cellulose, structural and thermal analysis, morphological characterization, sustainable materials

## Abstract

This study investigates the effect of *Eucomis autumnalis* (EA) cellulose on the structural, thermal, and crystallization behaviour of polybutylene succinate (PBS) and polycaprolactone (PCL) composites. X-ray diffraction (XRD) results showed that in both matrices, EA cellulose promoted nucleation, as indicated by increased peak intensity, while differential scanning calorimetry (DSC) showed reduced melting enthalpy, suggesting the formation of smaller, less perfect crystals. In PBS composites, EA cellulose acted as a crystallization disruptor, reducing crystallinity and enthalpy. Moreover, it slightly lowered the melting temperature. This is because EA cellulose contains β-(1→4) glycosidic bonds, which introduce –O– (ether) linkages along its polymer backbone. These linkages allow for a degree of rotational flexibility. When the cellulose is incorporated into PBS, this structural characteristic may contribute to a reduction in *T_m_*, likely by disrupting the crystallization of PBS chains. At 1 wt.% EA cellulose, broader, more intense melting peaks indicated imperfect crystal formation, while higher loadings (3 and 5 wt.%) resulted in narrower, less intense peaks, reflecting reduced crystallinity. These results are consistent with cooling-curve results and SEM images showing structural irregularities. In PCL composites, EA cellulose similarly reduced crystallinity and enthalpy without significantly affecting melting or crystallization temperatures. The decrease in the melting enthalpy from 55.6 J/g to 47.6 J/g suggested the formation of thinner lamellae and less organized crystals, a conclusion supported by stable crystallization temperatures and declining peak intensities in cooling curves. The combination of XRD and DSC data highlighted the dual role of EA cellulose: it enhances nucleation while hindering crystal growth, leading to the formation of more amorphous structures in both PBS and PCL matrices. These findings offer valuable insights into the potential use of EA cellulose as a functional modifier to tailor the properties of biopolymer composites for environmentally friendly, biodegradable applications.

## 1. Introduction

In recent years, there has been an increasing demand for polymeric materials due to their numerous advantages, including low cost, easy processability, durability, and erosion resistance [1]. However, the use of synthetic or non-biodegradable polymers has significantly contributed to atmospheric and environmental pollution. The main issue is that synthetic polymeric materials do not degrade but instead accumulate in wildlife habitats and aquatic environments after they have been disposed of [2]. The utilization of biodegradable polymers presents a compelling solution to the waste-disposal challenges associated with petroleum-based plastics because of their inherent biodegradability, biocompatibility, and environmental friendliness [3].

Among the various biodegradable polymers, polybutylene succinate (PBS) and polycaprolactone (PCL) stand out as highly promising aliphatic biodegradable polyesters due to their favourable properties, which include inherent biodegradability, biocompatibility, and environmental friendliness. Polybutylene succinate is synthesized from two monomers, succinic acid and 1,4-butanediol, and exhibits good biodegradability, thermal stability, and mechanical strength [4,5,6]. PCL is a semicrystalline biodegradable polymer formed through the ring-opening polymerization of ε-caprolactone [7]. It demonstrates excellent resistance to liquid solvents, as well as exceptional mechanical and chemical properties, making it more suitable for diverse commercial applications [8]. To further enhance the properties of these two polymers, PBS and PCL, the introduction of natural fillers, including cellulose, has been explored [9,10,11]. Cellulose is regarded as the most abundant organic biopolymer on earth and has distinctive properties. It can be acquired from various sources, including plants, wood cell walls, and algae tissues, as well as some species of bacteria [12,13]. Recent studies have focused on cellulose derived from medicinal plants as a reinforcement material because of its eco-friendly nature and availability [14]. *Eucomis autumnalis* (known as pineapple lily) is a native medicinal plant abundant in Africa. It is a monocot from the kingdom *Plantae*, family *Asparagaceae*, subfamily *Scilloideae*. The bulbs of this plant have been extensively used in traditional medicine for the treatment of a variety of diseases including respiratory issues, stomach aches, pain, fever, and inflammation [15,16]. Recent studies have shown a growing interest in polymer-based composites reinforced with cellulose derived from various medicinal plants [10,14,17]. For instance, studies by Sikhosana et al. [10] and Selikane et al. [18] have explored the use of *Eucomis autumnalis* cellulose in biopolymers such as PLA and PCL, respectively. These studies demonstrated that cellulose-reinforced composites exhibit significant improvements in stiffness and dimensional stability, making cellulose an ideal candidate for biopolymer enhancement. The extraction of cellulose from *Eucomis autumnalis* for use in biocomposites presents an opportunity to improve material performance and to support sustainable practices by using locally available, biodegradable plant resources.

This study explores the effect of *Eucomis autumnalis* (EA) cellulose on PBS and PCL by analysing the structural, morphological, and thermal properties of composites. By incorporating EA cellulose into PBS and PCL matrices, we aim to provide comprehensive insights into the interactions between cellulose and these biopolymers. The findings contribute to the growing body of research on cellulose-based biocomposites, highlighting their potential for use in the development of sustainable and biodegradable materials.

## 2. Materials and Methods

### 2.1. Materials

#### 2.1.1. *Eucomis autumnalis*

**Figure 1:**
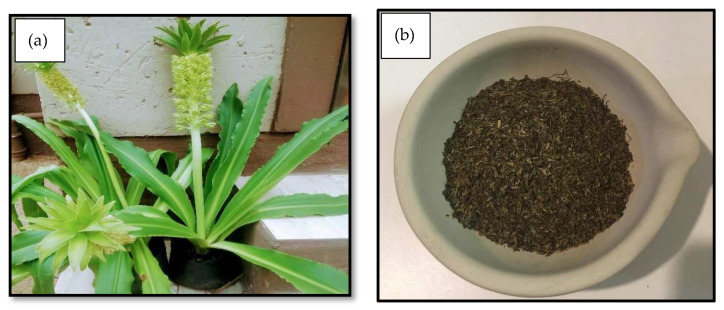
(**a**) A flowering *E. autumnalis* plant; (**b**) fine powder of dried *E. autumnalis* leaves [10]. [Open access].

*Eucomis autumnalis* (EA) plants were collected from the District of Maseru, in Lesotho, a country surrounded by South Africa. The taxonomy of this plant shows that it belongs to the kingdom Plantae, family *Asparagaceae*, genus *Eucomis*. Figure 1a shows a flowering *E. autumnalis* plant. Only the leaves were used to prepare cellulose in this project.

##### Extraction of Cellulose from *Eucomis autumnalis* Plant Leaves

To prepare the plant material, the leaves were stripped, then washed with distilled water and oven-dried at 40 °C for 3 weeks. The dried leaves were then ground into a fine powder using a blender. Figure 1b shows the fine powder of dried *E. autumnalis* leaves.

##### Delignification and Hemicellulose Removal

The fine powder of dried *E. autumnalis* leaves was bleached using 0.7% (*w*/*v*) sodium chloride solution. The pH of this mixture was then adjusted to pH 4 using 5% acetic acid solution. To further remove lignin, the mixture was heated at 100 °C in a water bath for 5 h, then washed with distilled water. The residuals from this mixture were further heated in 250 mL of 5% sodium sulphite solution in a water bath at 100 °C for 5 h, then thoroughly washed with distilled water to completely remove lignin [10].

##### Isolation of Cellulose

The residues from the previous step (delignification) were further treated with 250 mL of 18% (*w*/*v*) sodium hydroxide in a water bath set at 100 °C for 5 h to remove hemicellulose. After filtration, the resultant product was washed with distilled water and air-dried. To further remove other impurities, the recovered cellulose was further treated with 50 mL of dimethyl sulfoxide (DMSO), washed with distilled water, and then air-dried. Table 1 shows the chemical composition of cellulose extracted from *E. autumnalis* leaves. It shows the mass of the ground leaf powder, the mass of cellulose extracted, and the percentage yield of cellulose. From 60.02 g of ground leaf powder, 23.02 g of cellulose were extracted, for a 38% cellulose yield. This indicates that 38% of the ground leaf material is composed of cellulose. The purity of EA cellulose was assessed using FTIR and TGA, as described in Section 3.1.1 and Section 3.1.2, respectively.

#### 2.1.2. Poly(Butylene Succinate) (PBS)

Commercial poly (butylene succinate) (PBS) pellets extended with 1,6-diisocynatohexane was purchased from Sigma-Aldrich in Johannesburg, South Africa. PBS has a density of 1.3 g cm^−3^, a melting temperature of 120 °C, and a weight-average molecular weight of 63,000 g/mol [19].

#### 2.1.3. Poly(ε-Caprolactone) (PCL)

CapaTM 6500 polycaprolactone (PCL) pellets were purchased from Southern Chemicals in Johannesburg, South Africa. PCL has a density of 1.10 g cm^−3^, a glass transition temperature of −61 °C, a melting temperature of 60 °C, a degree of crystallinity of 35.7%, and a weight-average molecular weight of 113,400 g/mol [19].

### 2.2. Sample Preparation

All the samples (neat PBS, neat PCL, their blends, and blend composites) were prepared via solution casting using chloroform (from a consumable and chemical supplier; assayed purity 99.9%, density 1.49 g cm^−3^, and molecular weight 119.38 g mol^−1^). Chloroform was used because it dissolved both polymers at room temperature and evaporated easily. To prepare the thin films of neat PBS and PCL, a mass of 0.5 g of each polymer was weighed and dissolved in 15 mL of chloroform. The sample solution was stirred at room temperature for an hour then casted into evaporating dishes and left to dry for 4 h. The blends of PBS and PCL composites with different concentrations of cellulose (1, 3, and 5 wt.%) films were also prepared by combining different masses of each material to make a total mass of 0.5 g and dissolving the blends in 15 mL of chloroform. The same procedure of stirring the solution for an hour, casting it into the evaporating dishes, and drying for 4 h was also followed. To ensure uniform solvent evaporation, the films were vacuum-dried for 24 h at 40 °C to further remove residual solvent. Table 2 provides the different compositions of all the prepared samples.

### 2.3. Sample Characterization

#### 2.3.1. Fourier-Transform Infrared (FTIR) Spectroscopy

The samples were analysed using the Perkin-Elmer Spectrum 100 series spectrometer, manufactured by PerkinElmer, Inc., located in USA. It was fitted with a PIKE MiracleTM ATR and equipped with a diamond crystal. In this machine, the wavenumber was set over the range 450–4000 cm^−1^, and analyses were conducted using a resolution of 4 cm^−1^ and a total running of eight scans.

#### 2.3.2. X-Ray Diffraction (XRD)

X-ray diffraction analyses were done using a Bruker AXS D8 ADVANCED Discover diffractometer powder diffractometer with Cu Kα (1.5418 Å) radiation. The manufacturer of this equipment is in Germany. Continuous scans of the samples were carried out at 40 mA and 40 kV voltage using a locked-couple scan mode at a count interval of 1 s. The diffraction spectra were recorded in the 2θ range 20–80° with a scan rate of 0.02°/s.

#### 2.3.3. Scanning Electron Microscopy (SEM)

All prepared samples were surface-fractured using liquid nitrogen, and morphologies were studied using a JSM-7800F Ultra-High-Resolution Analytical Field Emission SEM, manufactured by JEOL Ltd., in Tokyo, Japan. The samples were coated with 5 nm iridium to ensure that the charge deposited on the surface of the sample by electron beam was earthed.

#### 2.3.4. Differential Scanning Calorimetry (DSC)

A heat flux Perkin Elmer Pyris 6000 differential scanning calorimeter, manufactured in Akron, OH, USA, was used to characterize the samples. The analysis was performed under nitrogen atmosphere flow (20 mL/min). All samples with a mass (~6.70 mg) were subjected to two heating cycles (from 0 to 130 °C) and one cooling cycle (from 130 to 0 °C) at a rate of 10 °C/min. The crystallization temperature, and melting temperature, as well as enthalpies, were determined from the first heating, cooling, and second heating scans. The degree of crystallinity was also calculated using Equation (1) (for neat polymers) and Equation (2) (for the composites), as follows:

(1) $$Xc\left(\%\right)=\left(\frac{\Delta Hm}{\Delta H^{0}m}\right)\times 100\%$$

(2) $$Xc=\left(\frac{\Delta Hm}{\Delta H^{0}m}\right)\times \frac{100\%}{\phi }$$

where *X_c_* is the degree of crystallinity, Δ*H_m_* is the melting enthalpy of the measured sample, Δ*H*^0^_*m*_ is the melting enthalpy of the 100% crystalline polymer, and *ϕ* is the weight fraction of a polymer in a blend or composite. Values of 200 J/g [20] and 139 J/g [8] were found for PBS and PCL, respectively.

#### 2.3.5. Thermogravimetric Analysis (TGA)

The thermal stabilities of the neat polymers, blends, and composites were measured on a Perkin-Elmer STA6000 thermogravimetric analyser, manufactured by Perkin-Elmer in Springfield, IL, USA. A mass of ~22 mg of each sample was heated from 30–650 °C under flow of nitrogen atmosphere (10 °C/min), and the mass loss % was recorded for each sample.

## 3. Results and Discussion

### 3.1. Structural Properties

#### 3.1.1. Fourier-Transform Infrared (FTIR) Spectroscopy

The results indicate that FTIR successfully identified the functional groups present in neat PBS, PCL, and EA cellulose, as well as in their various composites.

**Figure 2:**
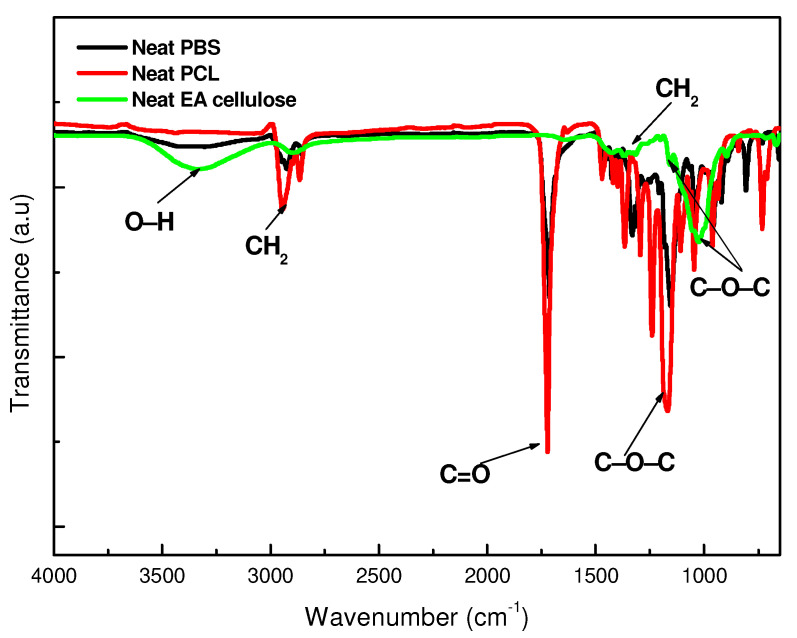
FTIR spectra of neat PBS, neat PCL, and neat EA cellulose.

For neat PBS, as shown in Figure 2 and summarized in Table 3, several characteristic peaks were observed. The initial peak at 3000–2845 cm^−1^ was assigned to C–H bond stretching and was followed by a sharp peak between 1713–1710 cm^−1^ attributed to the stretching vibration of the ester carbonyl group in the crystalline phases of PBS. The peaks at approximately 1264 and 1144 cm^−1^ correspond to the out-of-plane oscillations of CH_2_ and the symmetrical vibration of C–O–C bonds, respectively, which were also reported in similar studies [21,22]. Finally, the peak at 917 cm^−1^ corresponds to the C–OH bending of carboxylic acid groups in PBS, further confirming the structure. In the case of neat PCL, as depicted in Figure 2, characteristic peaks observed at 3000 and 2840 cm^−1^ were assigned to CH_2_ stretching vibrations. An intense sharp peak at around 1730–1715 cm^−1^ corresponds to C=O stretching. Additionally, the bands at 1293 and 1240 cm^−1^ were assigned to C–C and C–O stretching in the crystalline and amorphous phases, respectively, while peaks at 1190 and 1170 cm^−1^ represent the asymmetric and symmetric stretching of C–O–C bonds. For neat EA cellulose, the IR spectrum displayed a broad peak around 4000–2995 cm^−1^ that was attributed to O–H stretching and indicated strong hydroxyl-group presence. The band at 2890 cm^−1^ was assigned to C–H stretching of hydrocarbons, and a peak at 1640 cm^−1^ indicated water absorption, as suggested by previous studies [23]. Bands at 1162 and 1022 cm^−1^ corresponded to C–O–C ether stretching, characteristic of EA cellulose.

**Figure 3:**
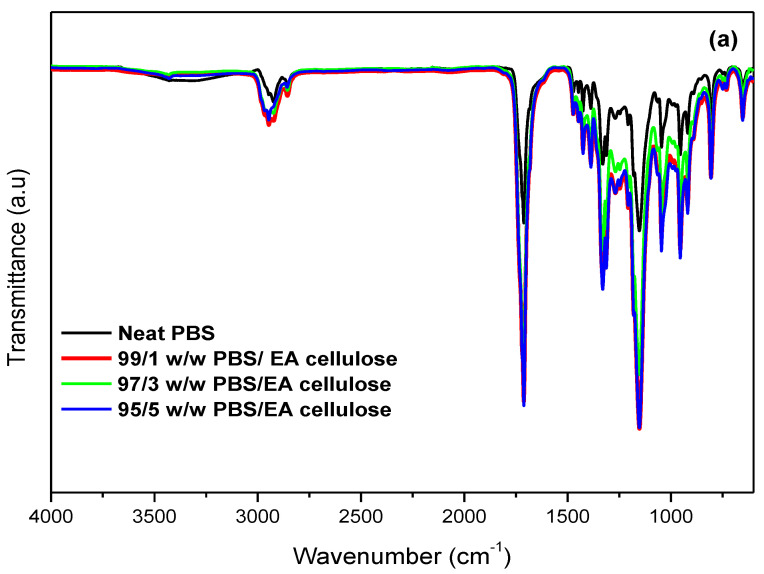

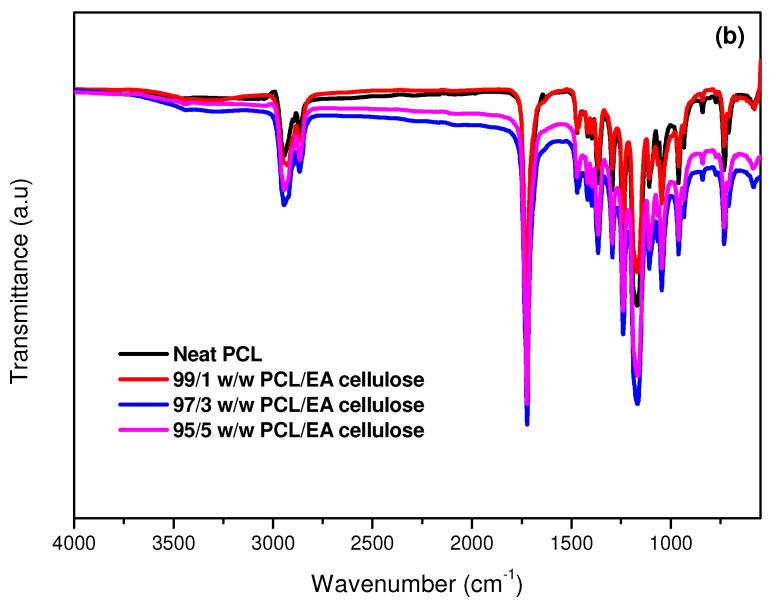
FTIR spectra of (**a**) neat PBS and its composites with EA cellulose; (**b**) neat PCL and its composites with EA cellulose at 1, 3, and 5 wt.%.

In Figure 3a, the FTIR spectra of neat PBS and its composites with EA cellulose (1, 3, and 5 wt.%) show that for neat PBS, peaks at 2926 cm^−1^ and 1711 cm^−1^ corresponded to C–H and carbonyl stretching, respectively, while peaks at 1327 and 1158 cm^−1^ were attributed to CH_2_ stretching and C–O–C vibration. The composites displayed an increase in peak intensity around 1711 and 1158 cm^−1^, suggesting enhanced interaction between EA cellulose and the less crystalline PBS matrix. These findings are consistent with the hypothesis that the hydroxyl groups of EA cellulose interact favourably with the amorphous regions of PBS, allowing for stronger interfacial adhesion [24].

In Figure 3b, the FTIR spectra of neat PCL and its composites with EA cellulose show that the PCL samples exhibited a characteristic peak around 1730–1715 cm^−1^ for C=O stretching, with additional peaks at 1364 and 1398 cm^−1^ for CH_2_ bending and at 1291 and 1179 cm^−1^ for C–C and C–O stretching, respectively. All the composites of PCL showed spectral features similar to those of neat PCL, with no noticeable changes upon the addition of EA cellulose. This suggests that the addition of EA cellulose does not significantly change the PCL structure. The nonsignificant changes observed in PCL composites suggest limited interaction between EA cellulose and the PCL matrix due to poor dispersion. Alternative processing techniques such as melt blending or the addition of a compatibilizer could improve compatibility.

#### 3.1.2. X-Ray Diffraction (XRD)

**Figure 4:**
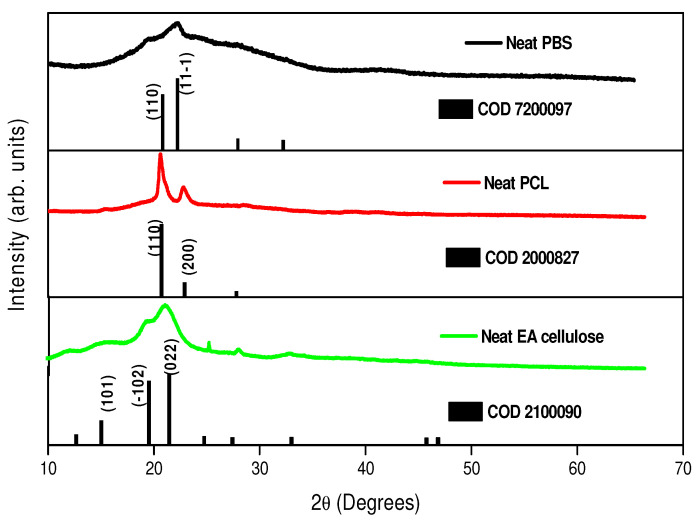
XRD patterns of neat PBS, neat PCL, and neat EA cellulose.

XRD was used to study the crystal structure of the prepared samples. As shown in Figure 4, neat PBS exhibited a monoclinic crystal structure, with distinct diffraction peaks at 2θ = ~20.6° and 23.2° representing the crystalline α phase. The broader peak at 44.0° corresponds to the amorphous phase of PBS. This indicates a higher proportion of amorphous content compared to crystalline content, consistent with findings from prior studies [5,25]. Analysis of neat PCL revealed an orthorhombic structure with strong, narrow peaks at 2θ = 21.5° and 23.9°, which are associated with higher crystallinity compared to neat PBS. Neat EA cellulose displayed characteristic peaks at 2θ = 15.4°, 20.9°, 24.6°, 27.6°, and 32.8°, which were attributed to a triclinic structure, with broad but intense peaks indicating a balance between the crystalline and amorphous phases.

**Figure 5:**
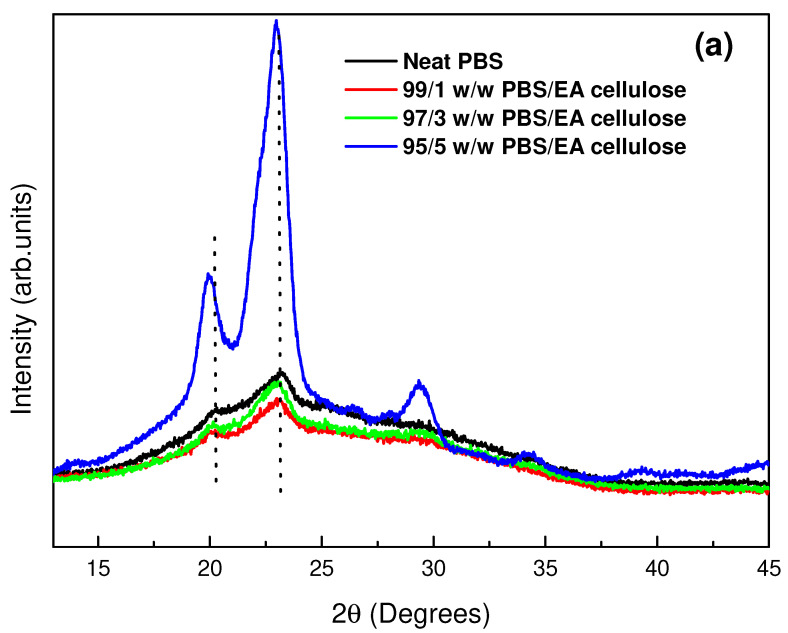

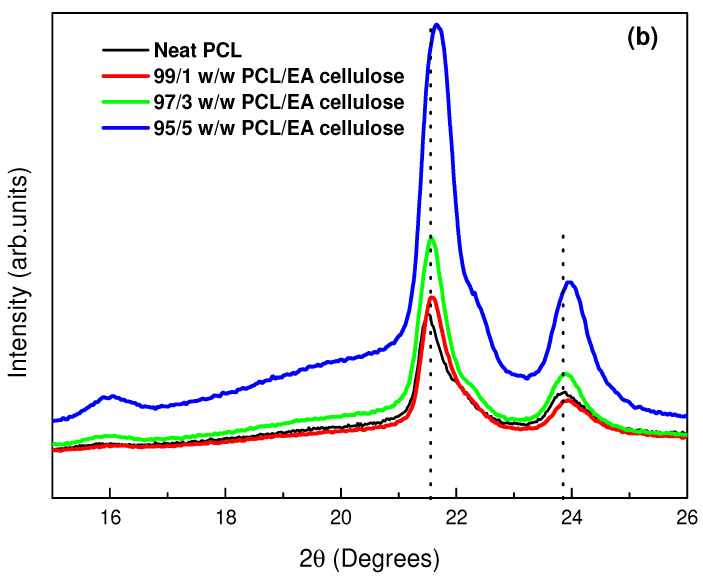
XRD patterns of (**a**) neat PBS and its composites with EA cellulose; (**b**) neat PCL and its composites with EA cellulose at (1, 3, and 5 wt.%).

Figure 5a shows neat PBS and its composites with EA cellulose at varying loadings (1, 3, and 5 wt.%). The data indicate observable shifts in diffraction peaks together with changes in peak intensity. The 1 wt.% and 3 wt.% EA cellulose composites exhibited lower peak intensities compared to neat PBS, indicating a reduction in crystallinity. This suggests an increase in the unit cell spacing, indicating a more disordered molecular arrangement that led to the formation of a greater proportion of amorphous regions within the PBS matrix. Such behaviour is often attributed to cellulose particles disrupting the crystalline structure of the polymer and thus hindering chain packing. The ether (-O-) linkages in EA cellulose contribute to increased chain flexibility, which can hinder efficient PBS chain packing and lead to longer unit cell spacing. At 5 wt.% EA cellulose loading, the peak intensity increased greatly and distinct crystalline peaks re-emerged. This shows that beyond a certain concentration, EA cellulose particles may act as nucleating agents, promoting polymer-chain mobility and facilitating crystalline formation. The enhanced crystallinity at higher EA cellulose content aligns with the previously published literature [26,27], which proposes that cellulose encourages the formation of ordered regions within the polymer matrix, especially when it is present in sufficient amounts. Figure 5b presents the diffraction patterns of neat PCL and its composites. The two primary peaks of PCL, observed at 2θ = 21.5° and 23.9°, shifted slightly to higher angles and increase in intensity with increased EA cellulose content. This shift corresponds to decreased unit cell spacing within the PCL matrix. The increase in peak intensity suggests enhanced crystallinity due to the EA cellulose acting as a nucleating agent and promoting more ordered polymer-chain alignment and crystal formation.

### 3.2. Morphological Properties

#### Scanning Electron Microscopy (SEM)

**Figure 6:**
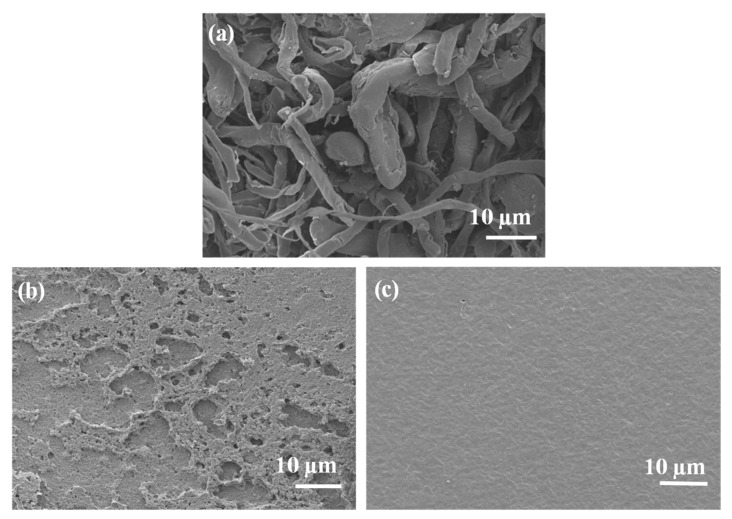
SEM images of (**a**) neat EA cellulose, (**b**) neat PBS, and (**c**) neat PCL.

Morphological analysis is important for understanding the interactions between the components in polymer composites. In this study, SEM was used to examine the morphology of the prepared samples. The SEM images of neat and blended materials are shown in Figure 6. Neat EA cellulose (Figure 6a) displays a fibrous morphology, which is consistent with similar observations reported by Selikane et al. [18]. The SEM image of neat PBS (Figure 6b) shows a rough, porous surface. This morphology agrees with findings by Asif et al. [28], who observed similar features in PBS films produced using the solvent casting method. The neat PCL sample (Figure 6c) exhibited a smooth surface without any pores, which is typical for PCL.

**Figure 7:**
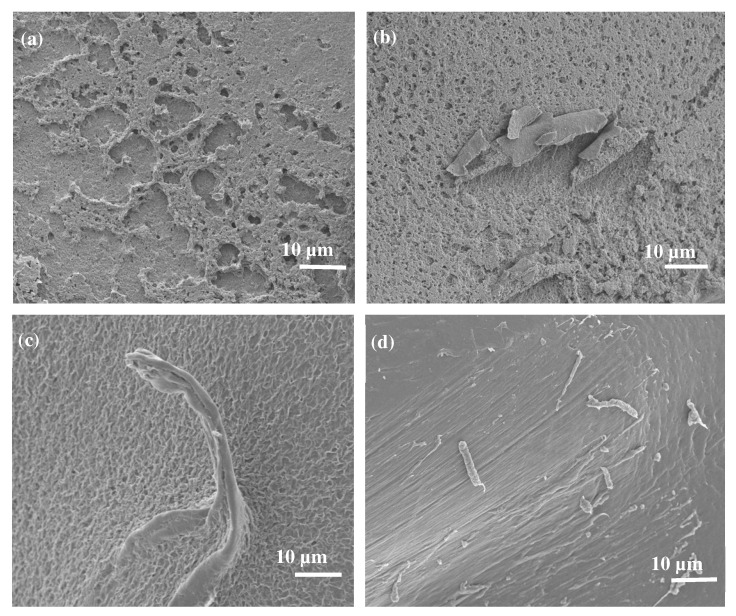
SEM images of (**a**) neat PBS, (**b**) 99/1 *w*/*w* PBS/EA cellulose, (**c**) 97/3 *w*/*w* PBS/EA cellulose, and (**d**) 95/5 *w*/*w* PBS/EA cellulose.

Further morphological studies were conducted on PBS/EA cellulose composites, as shown in Figure 7. SEM images of neat PBS (Figure 7a) revealed a porous surface. However, when EA cellulose was added at 1 wt.% (Figure 7b), the EA cellulose seemed to occupy the pores, making the surface of the PBS smoother. As the content of EA cellulose increased to 3 wt.% (Figure 7c), the fibres became clearly visible and were well-dispersed within the PBS matrix, further reducing the number of visible pores. At 5 wt.% of EA cellulose (Figure 7d), the dispersion of the cellulose fibres was even more pronounced, with the fibres well-distributed and the surface pores almost completely closed. This finding of improved dispersion and reduced pore size suggests that EA cellulose has a good compatibility with PBS due to the hydrophilicity of PBS. This further indicates a good interaction between PBS and EA cellulose, as observed in the FTIR results (Figure 3a).

**Figure 8:**
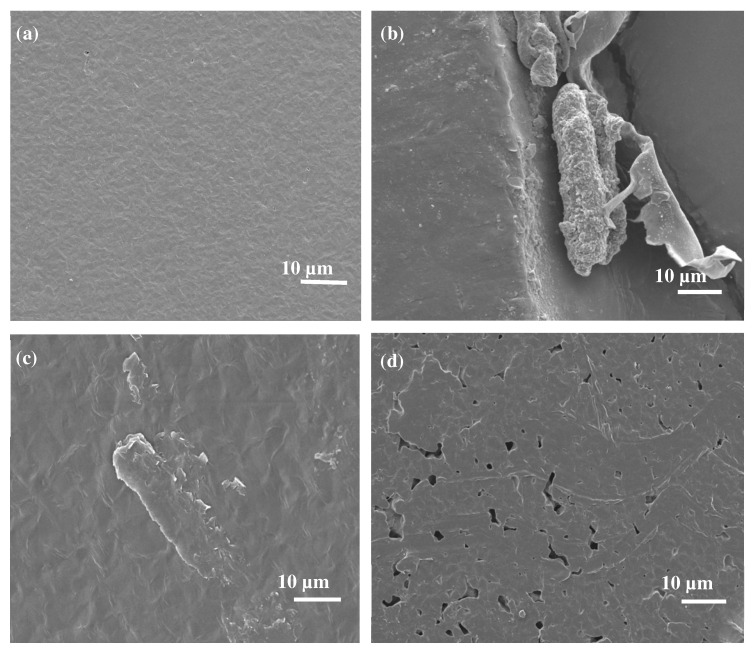
SEM images of (**a**) neat PCL, (**b**) 99/1 *w*/*w* PCL/EA cellulose, (**c**) 97/3 *w*/*w* PCL/EA cellulose, and (**d**) 95/5 *w*/*w* PCL/EA cellulose.

In the case of PCL/EA cellulose composites, as shown in Figure 8, neat PCL (Figure 8a) still exhibited a smooth surface. With the addition of 1 wt.% EA cellulose (Figure 8b), the morphology of PCL remained largely unchanged, but the cellulose appeared to be situated on the surface of the PCL, indicating poor adhesion between the two components. At higher EA cellulose contents of 3 wt.% (Figure 8c) and 5 wt.% (Figure 8d), the SEM images revealed the formation of cracks and pores in the PCL matrix. At 5 wt.% EA cellulose, a sea-island morphology was observed; this was attributed to the pulling out of the cellulose fibres, which left pores and scratches in the PCL surface. These results indicate poor interaction between PCL and EA cellulose. Selikane et al. [18] also reported similar findings.

### 3.3. Thermal Properties

#### 3.3.1. Differential Scanning Microscopy (DSC)

**Figure 9:**
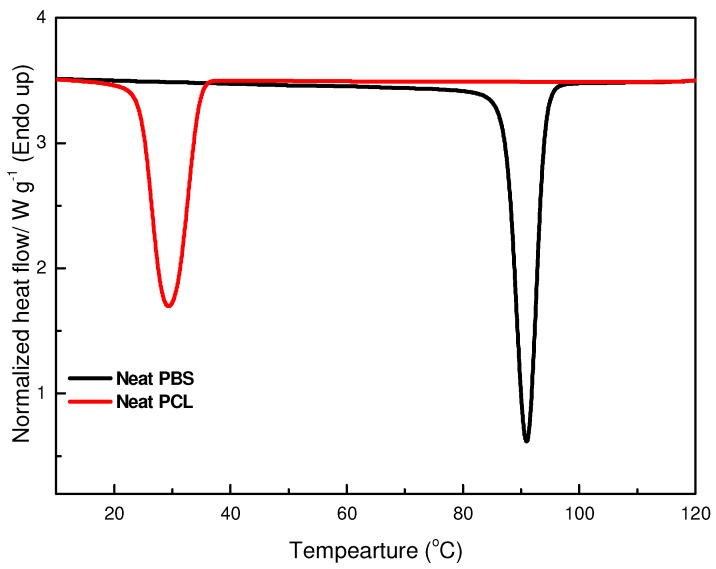
DSC cooling curves of neat PBS and PCL.

**Figure 10:**
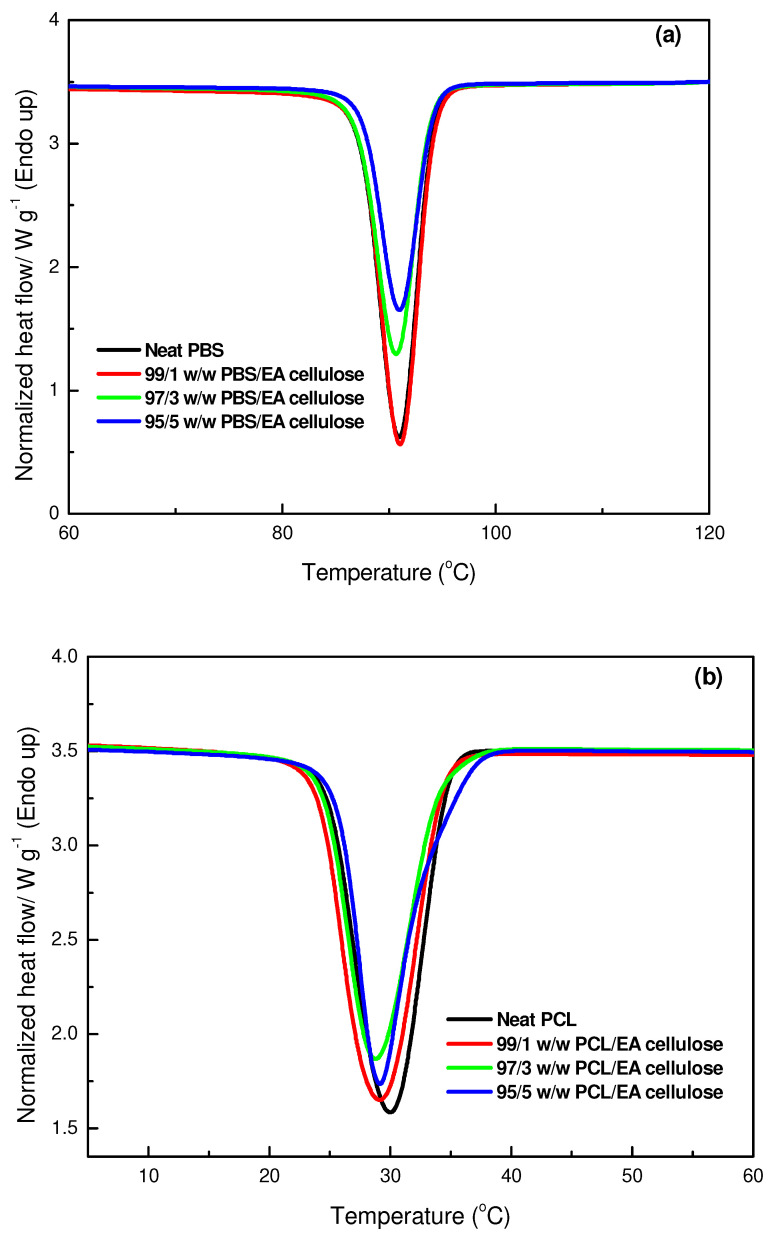
DSC cooling curves of (**a**) neat PBS and its composites with EA cellulose; (**b**) neat PCL and its composites with EA cellulose at 1, 3 and 5 wt.%.

The evaluation of the thermal properties of polymer blend systems is vital in understanding various transitions within these materials. DSC was used to investigate the thermal characteristics of the prepared samples, with a focus on key transitions such as melting temperature and crystallization temperature and on their associated enthalpies. The DSC cooling curves presented in Figure 9 and Figure 10, as well as in Table 4, show the crystallization behaviour of neat PBS and neat PCL. Neat PBS exhibits a crystallization temperature (*T_c_*) around 90.9 °C, while PCL crystallizes at a lower temperature, approximately 29.8 °C.

Figure 10a,b shows the impact of EA cellulose on the cooling curves of PBS and PCL. For PBS and its composites with EA cellulose, the crystallization temperature remained consistent, but the intensity and enthalpy of crystallization decreased as EA cellulose content increased (from −70.1 J/g to −55.6 J/g). In Figure 10b, the cooling curves for PCL and its composites with EA cellulose show no significant shift in crystallization temperature, which remained relatively stable. However, a decrease in peak intensities and crystallization enthalpies occurred, with no clear order based on EA cellulose content. This suggests that although XRD indicates the increased formation of crystalline regions, EA cellulose also disrupts the crystallization process during cooling by interfering with the growth of larger, well-formed spherulites, reducing the overall enthalpy of crystallization. Furthermore, the contradiction suggests that the EA cellulose promotes the formation of more nucleation sites, leading to the development of a greater number of smaller, imperfect crystalline regions rather than a lesser number of larger crystals. This can in turn increase XRD peak intensity because of the presence of more scattering centres while decreasing the crystallization enthalpy observed in DSC, which shows less energy released during the formation of crystals.

**Figure 11:**
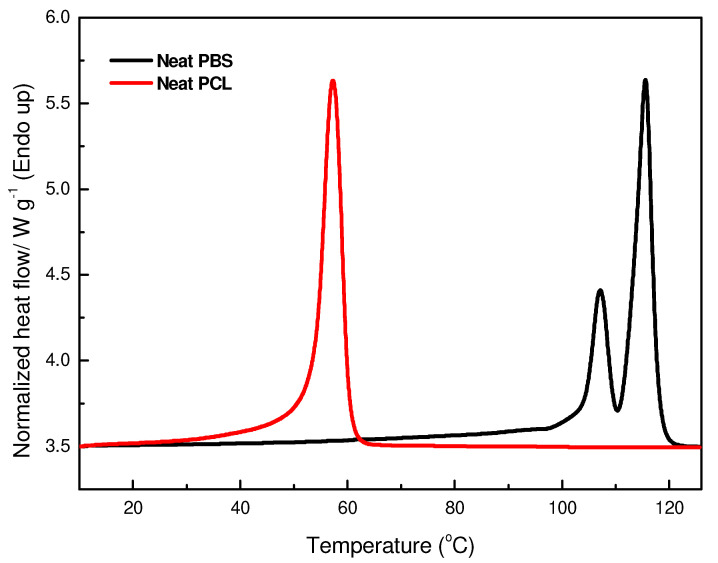
DSC second heating curves of neat PBS and neat PCL.

**Figure 12:**
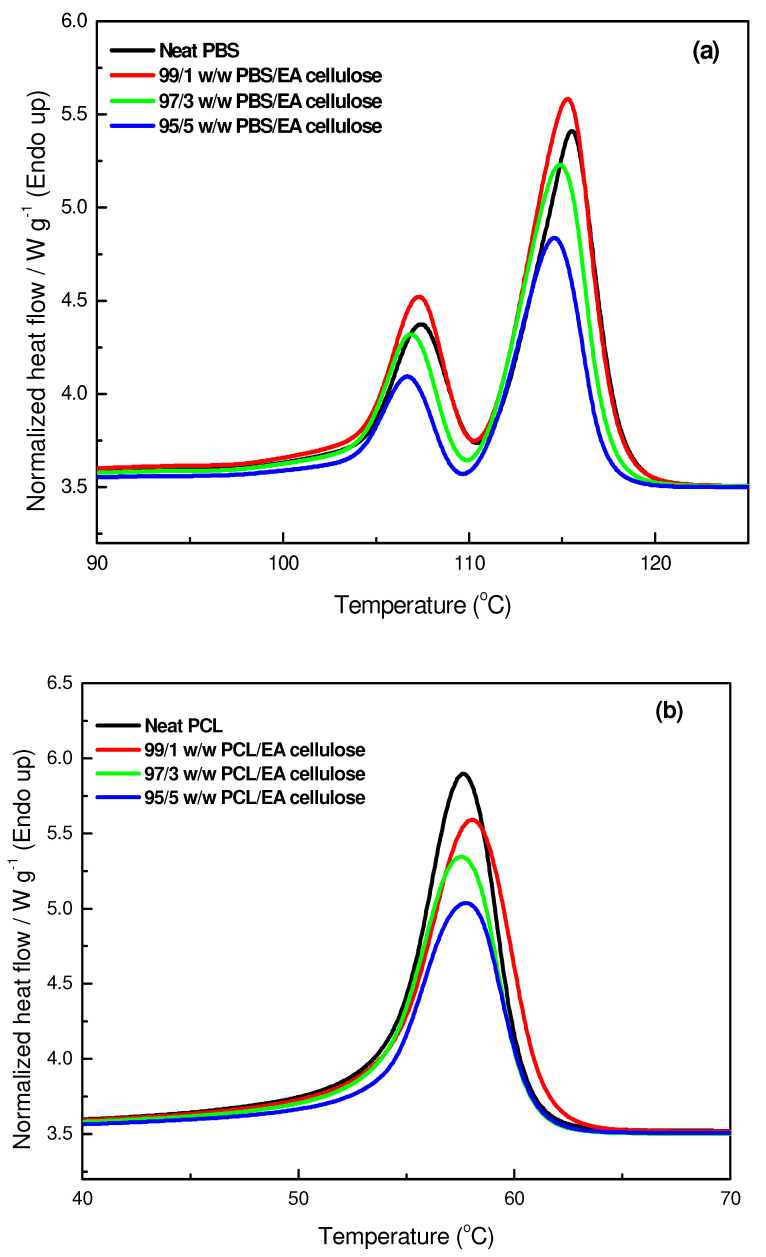
DSC second heating curves of (**a**) neat PBS and its composites with EA cellulose (**b**); neat PCL and its composites with EA cellulose at 1, 3, and 5 wt.%.

Figure 11 and Figure 12 show the second heating curves of neat PBS, PCL, and their composites with EA cellulose. The DSC thermograms of neat PBS and PCL are shown in Figure 11. For neat PBS, two melting peaks were observed. This indicates the presence of a crystalline structure that forms regions with varying degrees of organization. The first melting peak, occurring at approximately 107.4 °C, corresponds to the melting of smaller, less ordered crystallites, while the second peak at around 115.4 °C was attributed to the melting of larger, more ordered crystalline regions. This observation suggests the heterogeneity of the crystalline structure in PBS. Yoo et al. [29] reported similar observations of double melting peaks for PBS, attributing the first peak to the melting of the original crystallites and the second peak to the melting of recrystallized ones. The enthalpies for these peaks were found to be 12.4 J/g and 35.5 J/g, respectively, with a calculated degree of crystallinity (*X_c_*) of 31.6%. In contrast, neat PCL displayed only a single melting peak at 57.6 °C, with a higher degree of crystallinity (40.0%) compared to PBS. This result was also confirmed by X-ray diffraction (XRD) analysis, which revealed that PCL was more crystalline than PBS.

In Figure 12a, the DSC second heating curves of neat PBS and its composites with EA cellulose at various loadings (1, 3, and 5 wt.%) are shown. The addition of EA cellulose to PBS resulted in a slight decrease in the melting temperature of PBS for all composites. According to R.J. Young and P.A. Lovell [30], the stiffness of a polymer chain is primarily determined by the ease of rotation around its chemical bonds. The incorporation of certain linking groups, such as –O– (ether) or –CO–O– (ester) bonds, generally enhances chain flexibility and lowers the melting temperature (*T_m_*). In contrast, rigid structural units like p-phenylene increase stiffness and significantly raise *T_m_*. Since EA cellulose contains β-(1→4) glycosidic bonds, which introduce –O– (ether) linkages along its polymer backbone (as observed in Figure 2 and Table 3), these linkages allow for a degree of rotational flexibility. When EA cellulose is incorporated into PBS, this structural characteristic may contribute to a reduction in *T_m_*, likely by disrupting the crystallization of PBS chains [30]. However, the addition of 1 wt.% of EA cellulose caused the PBS peaks to become more intense and broader. This indicates the formation of imperfect crystals due to EA cellulose disrupting chain packing. For the composites with 3 and 5 wt.% EA cellulose, the peaks became narrower but less intense and the enthalpy and *X_c_* decreased with increasing EA cellulose content. This is in line with the cooling-curve results (Figure 10a), where crystallization temperature remained unchanged, but the crystallization enthalpy decreased with increasing EA cellulose content. The SEM images in Figure 7 also support this conclusion in that they show structural irregularities consistent with reduced crystallinity. Shi et al. [24] reported similar findings, noting a decrease in *T_m_* and Δ*H_m_* of PBS upon the addition of EA cellulose triacetate (CT). This was attributed to the restriction of molecular movement and hindrance of crystallization caused by CT. For neat PCL and its composites with EA cellulose (1, 3, and 5 wt.%), the DSC second heating curves are shown in Figure 12b. The results reveal that the presence of EA cellulose did not significantly affect the melting temperature of PCL. However, there was a noticeable decrease in the intensities, enthalpies, and degrees of crystallinity as the content of EA cellulose increased. Studies [9,31,32] have reported similarly negligible effects of cellulose on the *T_m_* of PCL. The melting enthalpy of PCL decreased from 55.6 J/g to 47.6 J/g with increasing loadings of EA cellulose, indicating that EA cellulose reduces the lamellar thickness of PCL crystals, resulting in the formation of less perfect crystals. This observation mirrors the cooling-curve results presented in Figure 10b, where crystallization temperature remained unchanged, but the peak intensities and enthalpy decreased inconsistently.

#### 3.3.2. Thermogravimetric Analysis (TGA)

**Figure 13:**
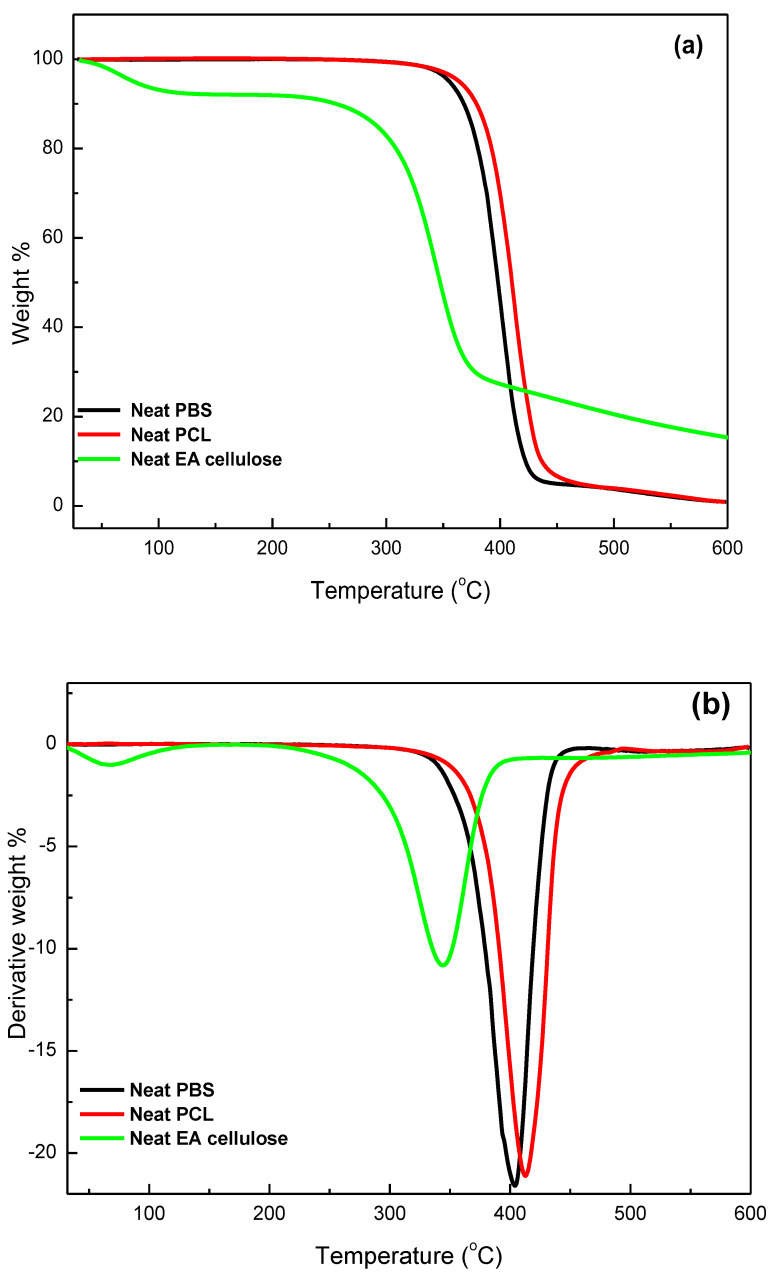
(**a**) TGA curves, (**b**) TGA derivative curves for neat PBS, PCL, and neat EA cellulose.

**Figure 14:**
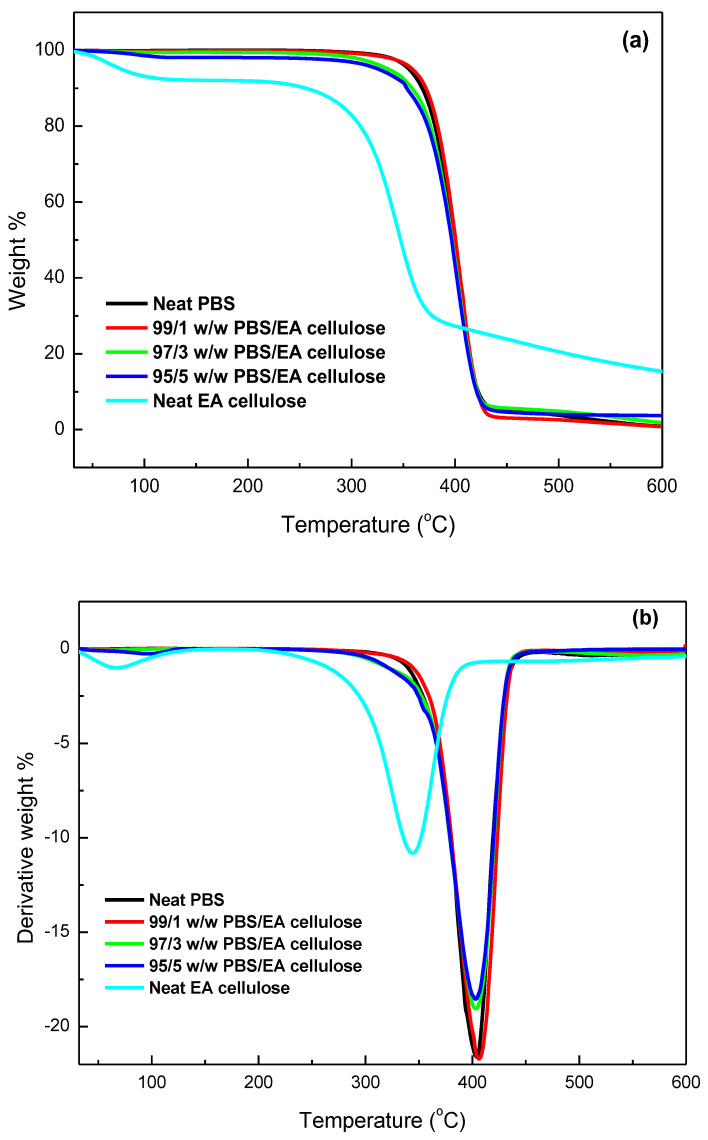
(**a**) TGA curves; (**b**) DTG curves for neat PBS and its composites with 1, 3, and 5 wt.% EA cellulose.

**Figure 15:**
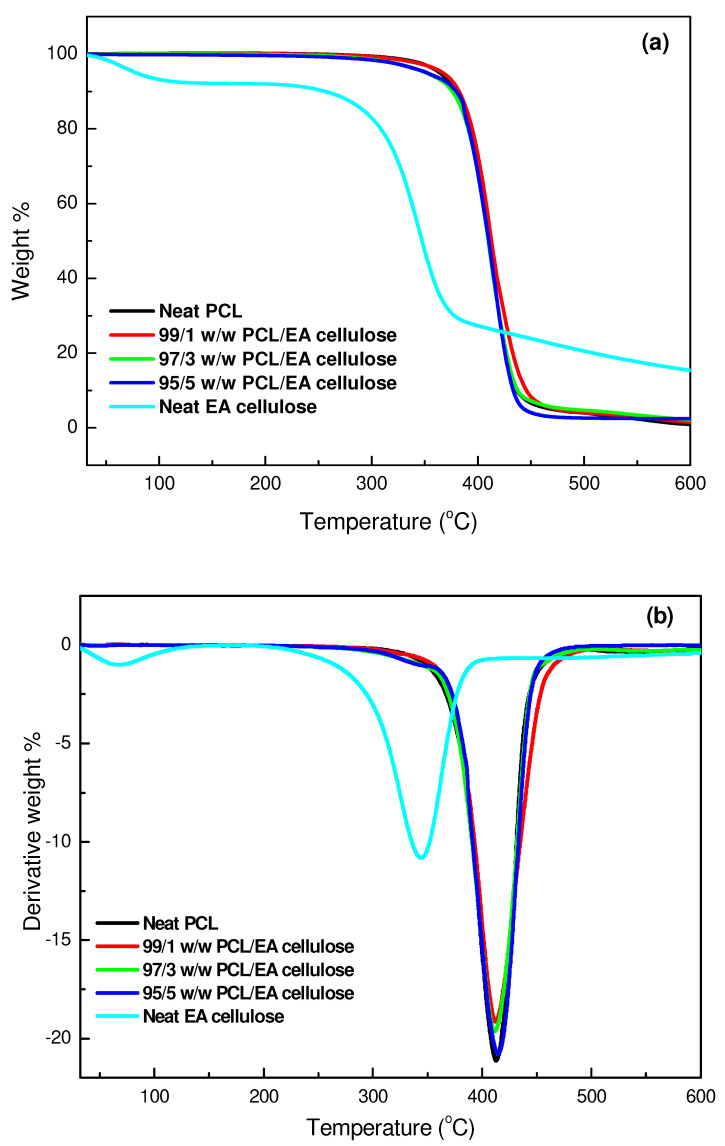
(**a**) TGA curves; (**b**) DTG curves for neat PCL and its composites with 1, 3, and 5 wt.% EA cellulose.

The thermal stability of the investigated samples was assessed using TGA, with a temperature range from 30 to 650 °C at a heating rate of 10 °C min^−1^. The results, as shown in Table 5 and Figure 13, Figure 14 and Figure 15, demonstrate the thermal degradation characteristics of various materials. Neat PBS begins to degrade at 347.9 °C and reaches its maximum decomposition temperature of 402.2 °C, with a residue of approximately 0.8%. This indicates that PBS has relatively low thermal stability compared to the other materials tested. Neat PCL, on the other hand, exhibits degradation onset at a higher temperature, 360.9 °C, with its maximum decomposition occurring at 413.2 °C and a residue of 0.9%, making it more thermally stable than PBS. In contrast, neat EA cellulose undergoes a two-step degradation process. The first step occurs between 28 and 100 °C and is attributed to the loss of absorbed and adsorbed water. The second degradation phase begins at 267 °C and extends to 344.4 °C, reflecting the breakdown of both cellulosic and non-cellulosic materials. The residue from EA cellulose is significantly higher, around 15%, highlighting its higher content of non-volatile materials.

PBS/EA cellulose and PCL/EA cellulose composites exhibit differing behaviour depending on the cellulose content. For example, in the PBS/EA cellulose composites (Figure 14), the addition of EA cellulose reduces the thermal stability, especially at higher loadings of 3 and 5 wt.%. These concentrations disrupt the movement of polymer chains within PBS, leading to reduced crystallinity and thermal stability. Similarly, the PCL/EA cellulose (Figure 15) composites behave similarly, with the 1 wt.% EA cellulose composite showing improved thermal stability over neat PCL, while higher cellulose loadings (3 and 5 wt.%) result in decreased thermal stability due to the disruption of the crystalline structure of PCL.

## 4. Conclusions

This study provides valuable insights into the structural, thermal, and morphological properties of PBS/EA cellulose and PCL/EA cellulose composites. The addition of EA cellulose into PBS and PCL matrices influences their crystallization behaviour, thermal properties, and structural organization. For PBS composites, EA cellulose acted as a crystallization disruptor, decreasing the crystallinity and enthalpy while also lowering the melting temperature. This is because EA cellulose contains β-(1→4) glycosidic bonds, which introduce –O– (ether) linkages along its polymer backbone. These linkages allow for a degree of rotational flexibility. When EA cellulose is incorporated into PBS, this structural characteristic may contribute to a reduction in *T_m_*, likely by disrupting the crystallization of the PBS chain. The broader and more intense melting peaks at low EA cellulose content (1 wt.%) were an indication of the formation of imperfect crystals, while higher loadings (3 and 5 wt.%) resulted in narrower, less intense peaks, showing reduced crystallinity. This behaviour is in alignment with the cooling-curve results, which showed stable crystallization temperatures but decreasing crystallization enthalpy, a finding further supported by SEM images revealing structural irregularities. For PCL composites, EA cellulose similarly reduced crystallinity and enthalpy without significantly affecting the melting or crystallization temperatures. The decrease in melting enthalpy from 55.6 J/g to 47.6 J/g indicated thinner lamellae and less perfect crystal formation. The stable crystallization temperature, paired with decreasing peak intensity and enthalpy in both the heating and cooling curves, suggests that EA cellulose disrupted crystal growth while having little impact on nucleation. XRD data confirmed this result in that there was increased peak intensity, indicating the formation of more nucleation sites, but the resulting crystals were smaller and less ordered. Overall, EA cellulose played a dual role, promoting nucleation while inhibiting proper crystal growth, and thus led to the formation of more amorphous, less crystalline structures in both PBS and PCL matrices. This combination of effects supports its potential for use as a modifier to tailor the thermal and mechanical properties of biopolymer composites.

## Figures and Tables

**Table 1:** Extraction yield of cellulose from *E. autumnalis* leaf powder.

Sample	Mass of the Ground Powder (g)	Mass of Cellulose (g)	% Yield of Cellulose
Cellulose from the leaves	60.02	23.02	38

**Table 2:** Weight % of the components in the samples.

PBS	PCL	EA Cellulose
100	0	0
99	0	1
97	0	3
95	0	5
0	100	0
0	99	1
0	97	3
0	95	5

**Table 3:** FTIR analysis of neat PBS, PCL, and EA cellulose.

Materials	Assignment	Wavenumber (cm^−1^)
PBS	C-H stretching	2926
	C=O stretching vibration	1711
	-CH_2_ stretching	1327
	-C-O-C symmetrical vibration	1158
	C-OH bending	917
PCL	-CH_2_ stretching	3000–2840
	C=O stretching	1730–1715
	C-C and C-O stretching	1291 and 1179
	-C-O-C, asymmetrical and symmetrical	1190 and 1170
EA cellulose	-OH stretching	4000–2995
	C-H stretching	2890
	C-O-C stretching	1162–1022

**Table 4:** Summary of DSC data for all prepared samples.

SAMPLES	*T_m_*/°C	*∆H_m_*/J/g	*T_c_*/°C	*∆H_c_*/J/g	*X_c_*/%
Neat PBS	107.4 ^b^ ± 0.1 115.7 ^c^ ± 0.2	14 ^b^ ± 0.6 39 ^c^ ± 2.9	90.2 ^b^ ± 0.0	70 ^b^ ± 2.4	27.5 ^b^
99/1 *w*/*w* PBS/EA cellulose	107.3 ^b^ ± 0.1 115.5 ^c^ ± 0.2	15 ^b^ ± 0.8 41 ^c^ ± 1.6	92.0 ^b^ ± 0.3	72 ^b^ ± 2.5	28.4 ^b^
97/3 *w*/*w* PBS/EA cellulose	107.2 ^b^ ± 0.2 115.4 ^c^ ± 0.2	11 ^b^ ± 1.1 31 ^c^ ± 1.7	90.9 ^b^ ± 0.2	57 ^b^ ± 2.6	21.3 ^b^
95/5 *w*/*w* PBS/EA cellulose	106.9 ^b^ ± 0.2 114.9 ^c^ ± 0.1	12 ^b^ ± 1.3 32 ^c^ ± 3.0	91.0 ^b^ ± 0.2	56 ^b^ ± 1.4	23.0 ^b^
Neat PCL	57.6 ^a^ ± 0.2	56 ^a^ ± 2.6	29.8 ^a^ ± 0.4	68 ^a^ ± 1.8	40.0 ^a^
99/1 *w*/*w* PCL/EA cellulose	57.8 ^a^ ± 0.2	55 ^a^ ± 0.6	28.9 ^a^ ± 1.5	64 ^a^ ± 1.5	39.7 ^a^
97/3 *w*/*w* PCL/EA cellulose	57.4 ^a^ ± 0.2	51 ^a^ ± 4.7	28.6 ^a^ ± 0.2	62 ^a^ ± 4.2	38.0 ^a^
95/5 *w*/*w* PCL/EA cellulose	57.9 ^a^ ± 0.4	48 ^a^ ± 4.5	29.3 ^a^ ± 0.6	53 ^a^ ± 0.9	36.0 ^a^

*T_m_*—melting peak temperature, Δ*H_m_*—melting enthalpy, *T_c_*—crystallization temperature, Δ*H_c_*—crystallization enthalpy, *X_c_*—degree of crystallinity. Superscript a is for PCL peaks; b and c, respectively, represent the first and second peaks of PBS.

**Table 5:** Summary of TGA and DTG results for all investigated samples.

SAMPLES	*T_onset_*/°C	*T*_50%_/°C	*T_max_*/°C	Char/mass%
Neat EA cellulose	85.69 309.03	344.16	83.42 344.79	15.3
Neat PBS	347.85	397.03	402.17	0.8
99/1 *w*/*w* PBS/EA cellulose	374.78	401.03	406.16	0.2
97/3 *w*/*w* PBS/EA cellulose	372.13	397.01	403.81	1.4
95/5 *w*/*w* PBS/EA cellulose	371.79	394.25	403.08	4.2
Neat PCL	360.87	409.65	413.17	0.9
99/1 *w*/*w* PCL/EA cellulose	368.59	410.20	411.80	1.4
97/3 *w*/*w* PCL/EA cellulose	373.74	410.57	412.08	1.8
95/5 *w*/*w* PCL/EA cellulose	382.30	409.31	414.55	3.0

## Data Availability

Data required are available upon request.

## Abbreviations

The following abbreviations are used in this manuscript:

EA	Eucomis autumnalis
PBS	Poly (butylene succinate)
PCL	Poly(caprolactone)
FTIR	Fourier-transform infrared spectroscopy
XRD	X-ray Diffraction
SEM	Scanning electron microscopy
DSC	Differential scanning calorimetry
TGA	Thermogravimetric analysis
